# Hydrogen enhances strength and ductility of an equiatomic high-entropy alloy

**DOI:** 10.1038/s41598-017-10774-4

**Published:** 2017-08-29

**Authors:** Hong Luo, Zhiming Li, Dierk Raabe

**Affiliations:** Max-Planck-Institut für Eisenforschung, Max-Planck-Straße 1, Düsseldorf, 40237 Germany

## Abstract

Metals are key materials for modern manufacturing and infrastructures as well as transpot and energy solutions owing to their strength and formability. These properties can severely deteriorate when they contain hydrogen, leading to unpredictable failure, an effect called hydrogen embrittlement. Here we report that hydrogen in an equiatomic CoCrFeMnNi high-entropy alloy (HEA) leads not to catastrophic weakening, but instead increases both, its strength and ductility. While HEAs originally aimed at entropy-driven phase stabilization, hydrogen blending acts opposite as it reduces phase stability. This effect, quantified by the alloy’s stacking fault energy, enables nanotwinning which increases the material’s work-hardening. These results turn a bane into a boon: hydrogen does not generally act as a harmful impurity, but can be utilized for tuning beneficial hardening mechanisms. This opens new pathways for the design of strong, ductile, and hydrogen tolerant materials.

## Introduction

Metallic materials are the manufacturing backbone of our industrialized civilization owing to their excellent load bearing capacity, ductility and damage tolerance. They occupy key roles in providing sustainable engineering and manufacturing solutions to such diverse fields as energy supply, transportation, health, infrastructure and safety. This excellent mechanical behavior can drastically deteriorate when metals are exposed to certain elements. Since 1874 it is understood that the lightest of them all, hydrogen, can be dangerous as it causes catastrophic and unpredictable failure, a phenomenon referred to as hydrogen embrittlement^1^. All metallic alloys can suffer from it^2–6^, be it in engineering parts used in vehicles, planes or power plants or in the context of future fusion and hydrogen fuel and energy storage driven industries.

Although these threats and opportunities have motivated nearly one and a half centuries of intense research^7–11^, hydrogen remains not only a ubiquitous but also a threatening element in engineering metallic alloys. Once hydrogen has entered into metals it accumulates in voids and gets trapped at vacancies, dislocations and internal interfaces, i.e. at lattice defects which determine the physical, chemical and mechanical properties of metals^12–15^. Once occupying these sites, hydrogen damages the material through enhanced localized plasticity^4, 16^, decohesion^17^, vacancy stabilization^18, 19^, hydride formation^20, 21^ or void coalescence^22^.

Although practically all metals suffer from such phenomena, the high-entropy alloy (HEA) investigated here seems to be not only less prone to hydrogen embrittlement but it even profits from its presence. HEAs are a new class of materials originally defined as solid metallic solutions composed of five or more principal elements in equimolar or near-equimolar ratios for yielding high configurational entropy^23–26^. This concept introduces a new path for developing advanced materials with some unique mechanical properties^27–30^. The five-component equiatomic CoCrFeMnNi alloy, also referred to as Cantor’s alloy^31^, is one of the most appealing HEAs due to the high thermodynamic stability of its single face-centred cubic (f.c.c.) structure and the excellent mechanical properties under various temperatures^27^.

Owing to these features we picked this equiatomic CoCrFeMnNi model HEA for studying its changing mechanical tensile behaviour when exposed to hydrogen. We show that this material is not only resistant to hydrogen embrittlement but we even observe its beneficial role as alloying element as it improves rather than deteriorates both, the material’s strength and ductility. The key idea behind this turnaround lies in decreasing the stability of the f.c.c. lattice structure of the matrix via hydrogen alloying to trigger more intense nanotwinning upon loading, thereby improving strain-hardening of the alloy.

## Results and Discussion

### Starting microstructure

**Figure 1:**
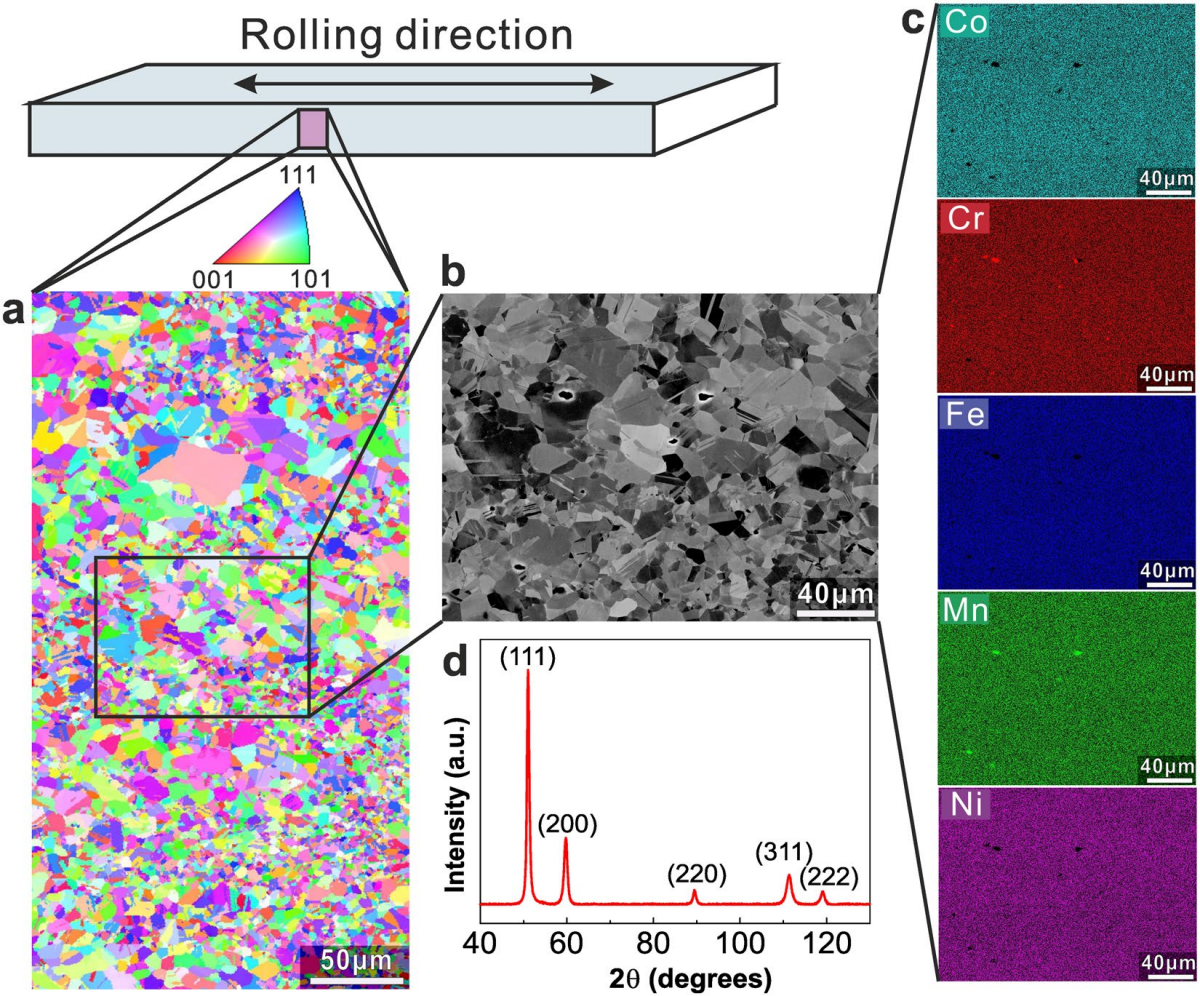
Microstructure and elemental distribution in the fully recrystallized equiatomic CoCrFeMnNi HEA. (**a)** Electron backscatter diffraction (EBSD) reveals the grain size to be in the range of 1~25 μm. (**b**) BSE image from the region marked in **a** confirms equiaxed grain structure. (**c)** EDS maps reveal the uniform distribution of all elements (i.e., Co, Cr, Fe, Mn, and Ni). (**d**) XRD pattern confirms the single f.c.c. phase structure.

The equiatomic CoCrFeMnNi HEA was produced by melting and casting in a vacuum induction furnace with high-purity elemental starting materials. The cast alloy was hot-rolled and homogenized followed by water-quenching. Cold-rolling and recrystallization annealing were performed to refine the grain size. Figure 1 shows the typical microstructure and elemental distribution in the CoCrFeMnNi HEA after recrystallization annealing. The material has a grain size in the range of 1–25 μm with random crystallographic texture (Fig. 1a,b). The back-scattered electron (BSE) image (Fig. 1b) and the corresponding energy-dispersive X-ray spectroscopy (EDS) maps (Fig. 1c) reveal that all elements (Co, Cr, Fe, Mn and Ni) are uniformly distributed. The X-ray diffraction (XRD) pattern in Fig. 1d confirms the f.c.c. phase structure.

### Strength and ductility upon hydrogen charging

**Figure 2:**
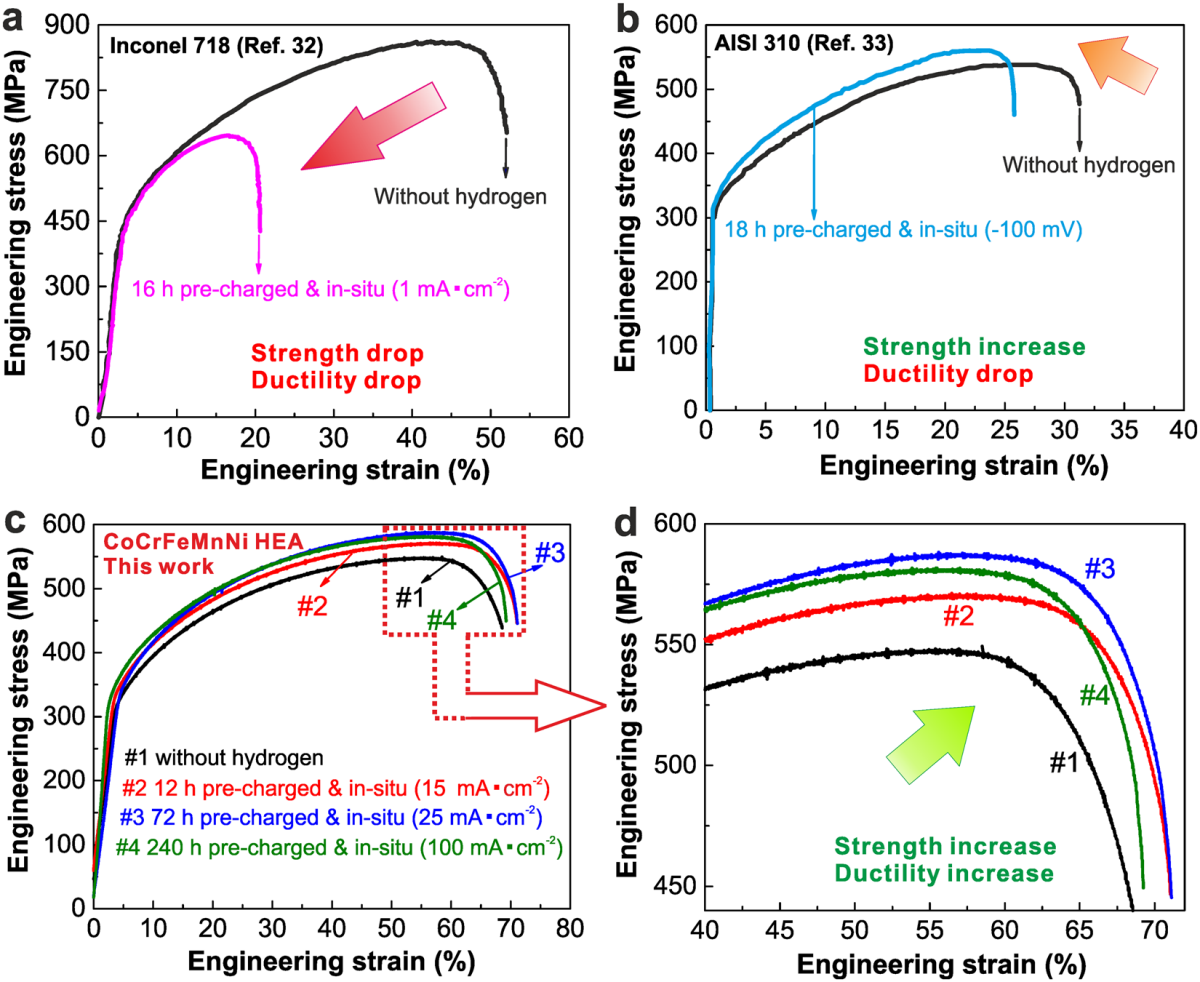
Tensile deformation behavior of different metals including the CoCrFeMnNi HEA under various *in-situ* hydrogen charging conditions. (**a**) Engineering stress-strain curves of nickel-based alloy Inconel 718 (56Ni-18Fe-18Cr-5Nb-3Mo) with and without hydrogen (16 h pre-charged and *in-situ* tested at 1 mA·cm^−2^ in 3 wt.% NaCl solution, strain rate of 1 × 10^−4^ s^−1^, data taken from ref. 32). (**b**) Engineering stress-strain curves of AISI 310 stainless steel (55Fe-25Cr-20Ni) with and without hydrogen (18 h pre-charged and *in-situ* tested at −100 mV in 0.5 M H_2_SO_4_ solution, strain rate of 1 × 10^−4^ s^−1^, data taken from ref. 33). (**c**) Engineering stress-strain curves of CoCrFeMnNi HEA under various *in-situ* hydrogen charging conditions (Supplementary Fig. 1) at a quasistatic strain rate of 1 × 10^−4^ s^−1^. Three samples were tested for each hydrogen charging condition. (**d**) Zoom-in image revealing the joint increases in ultimate tensile strength and elongation for the CoCrFeMnNi HEA. Average values of the ultimate tensile strength and total elongation as well as the corresponding standard deviations are summarized in the supplementary Table 1.

Figure 2 shows the tensile deformation behavior of different materials including the CoCrFeMnNi HEA under various *in-situ* hydrogen charging conditions (Supplementary Fig. 1). Figure 2a reveals that the alloy Inconel 718 (56Ni-18Fe-18Cr-5Nb-3Mo), a benchmark material for tolerance against hydrogen embrittlement, shows significant decrease in both strength and ductility after hydrogen charging^32^. The AISI 310 stainless steel (55Fe-25Cr-20Ni) shows an increase in ultimate strength but a decrease in total elongation when exposed to hydrogen (Fig. 1b)^33^. It has also been reported that hydrogen could be controlled to increase the ductility and yet reduce the flow stress of some intermetallic-based titanium alloys^34^. In line with these well studied example alloys literature confirms that the hydrogen induced decrease of strength and/or ductility are characteristic features common to metallic alloys while the simultaneous increase in both, strength and ductility has not been observed before.

The CoCrFeMnNi HEA studied here shows exactly this opposite trend when exposed to hydrogen as demonstrated in Fig. 1c,d. Curve #1 shows the tensile behavior of the CoCrFeMnNi HEA without hydrogen, revealing an ultimate tensile strength (UTS) of 545 MPa and a total elongation of ~68%. We then find that hydrogen charging in the current work does not cause any deleterious effects on the materials’ strength and ductility (curve #2~4). On the contrary, the alloy pre-charged for 12 h and *in-situ* tensile tested with a current density of 15 mA·cm^−2^ (curve #2) shows a notable increase of both, ultimate tensile strength (571 MPa) and uniform elongation (~71%) compared to the sample tested without any preceding hydrogen alloying. Note that the diffusible hydrogen concentration in the sample under this condition (12 h pre-charged at a current density of 15 mA·cm^−2^) was determined to be 8.01 wt.ppm according to the hydrogen desorption rate curves (Supplementary Fig. 2). Moreover, as the diffusible hydrogen concentration increased to 15.22 wt.ppm (72 h pre-charged at a current density of 25 mA·cm^−2^) (curve #3), the ultimate tensile strength and uniform elongation are 588 MPa and ~71%, respectively, which are ~7.9% and ~4.4% larger, respectively, than those observed for the same alloy without hydrogen (curve #1). With further increasing the diffusible hydrogen concentration to 33.25 wt.ppm (240 h pre-charged at a current density of 100 mA·cm^−2^), the HEA (curve #4) shows a slightly reduced increment in its ultimate strength and uniform elongation compared to the alloy with a hydrogen concentration of 15.22 wt.ppm (curve #3). This observation suggests that the induced hydrogen alloying, promoting both strength and ductility, assumes certain saturation states. Recent work by Zhao *et al*.^35^ showed that a gaseous hydrogen charged equiatomic HEA with a hydrogen concentration of ~76.5 wt.ppm exhibitts a slight decrease in elongation, although it showed resistance to gaseous hydrogen embrittlement that is superior to that of 304 and 316 L austenitic stainless steels. This observation further indicates that hydrogen induced enhancement of strength and ductility are strongly dependent on the hydrogen concentration in the material. This is related to the competition between the beneficial and the unfavorable mechanisms during the deformation of the hydrogen charged samples as discussed below.

We also find that the hydrogen alloying did not exert any significant effects on the yield strength of the equiatomic CoCrFeMnNi HEA (Fig. 2c). This means that hydrogen did not introduce observable solute strengthening in this alloy. The corresponding strain-hardening behavior with respect to the true strain in various hydrogen alloying conditions (Supplementary Fig. 3) indicates that over the entire deformation process, the hydrogen alloyed HEAs show higher strain-hardening rate than the reference material without hydrogen. Among the investigated conditions, the CoCrFeMnNi HEA with a hydrogen concentration of 15.22 wt.ppm (72 h pre-charged at a current density of 25 mA·cm^−2^) (curve #3) has the highest strain-hardening rate, which is consistent with the strength and elongation results shown in Fig. 2c,d.

### Fracture behavior upon hydrogen charging

**Figure 3:**
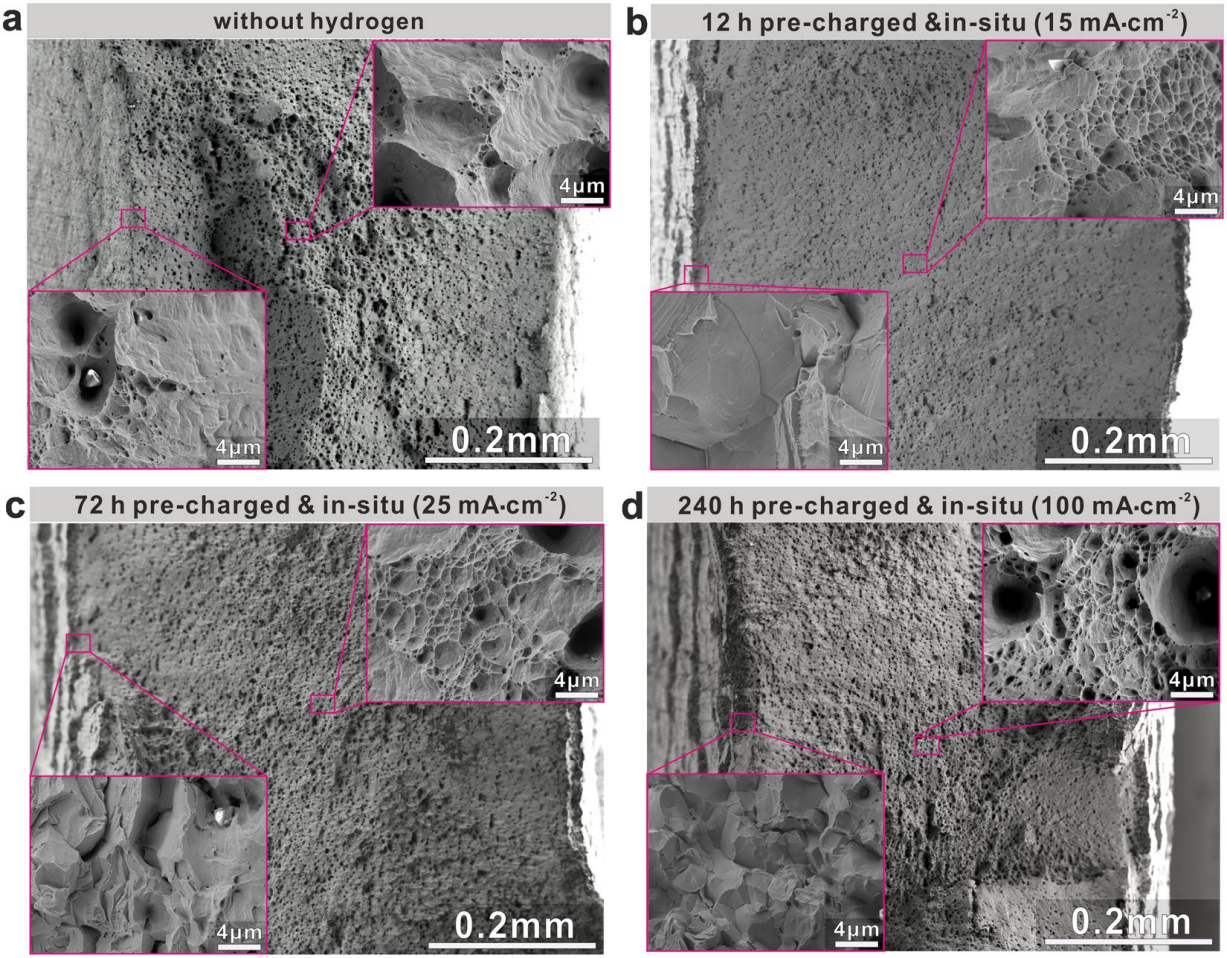
Images of fractured CrMnFeCoNi HEA samples after *in-situ* tensile testing under various hydrogen charging conditions. **(a**) Ductile dimpled fracture morphology for samples without hydrogen. (**b**,**c** and **d**) are images of samples fractured after *in-situ* tensile testing with hydrogen pre-charging conditions of 12 h at 15 mA·cm^−2^, 72 h at 25 mA·cm^−2^, and 240 h at 100 mA·cm^−2^, respectively.

To understand the fracture behavior of the hydrogen alloyed CoCrFeMnNi HEA, we studied the morphology of the fracture surfaces of all samples after *in-situ* tensile testing. Figure 3a shows a fully ductile fracture, characterized by the growth and coalescence of microvoids in both, edge and center regions of the sample without hydrogen alloying. Some particles, which had acted as typical initiation sites of microvoids, were found inside the voids on the fracture surface. The EDS results (Supplementary Fig. 4) show that they are enriched in Mn, Cr, S and Al. The hydrogen alloyed HEA samples also display the primary fracture mode of microvoid coalescence (Fig. 3b,c and d). However, also some intergranular fracture was observed on the edges of the hydrogen alloyed HEA samples.

Since hydrogenation in the present study was conducted by cathodic charging in alkaline solution and the tensile tests were performed under *in-situ* hydrogen charging conditions, a gradient in hydrogen concentration from the edge to the inner regions of the samples could have been induced according to previous studies on hydrogen embrittlement of other materials^4^. For this reason associated with the charging process, the intergranular fracture feature observed in the edges is attributed to the high hydrogen concentration near the edges of the samples during the tensile testing in alkaline solution. The fracture mode of microvoid coalescence in the inner regions suggests lower hydrogen concentration in these zones. We also observe that the very fine (<1 μm) ductile dimples in the inner regions of the fracture surface in the hydrogen alloyed samples (Figs. 3b,c and d) occur much more frequently compared to the reference alloy without hydrogen (Fig. 3a). This might be related to the more extensively occurring nanotwinning in the hydrogen alloyed HEAs discussed below.

### Deformation mechanism upon hydrogen charging

**Figure 4:**
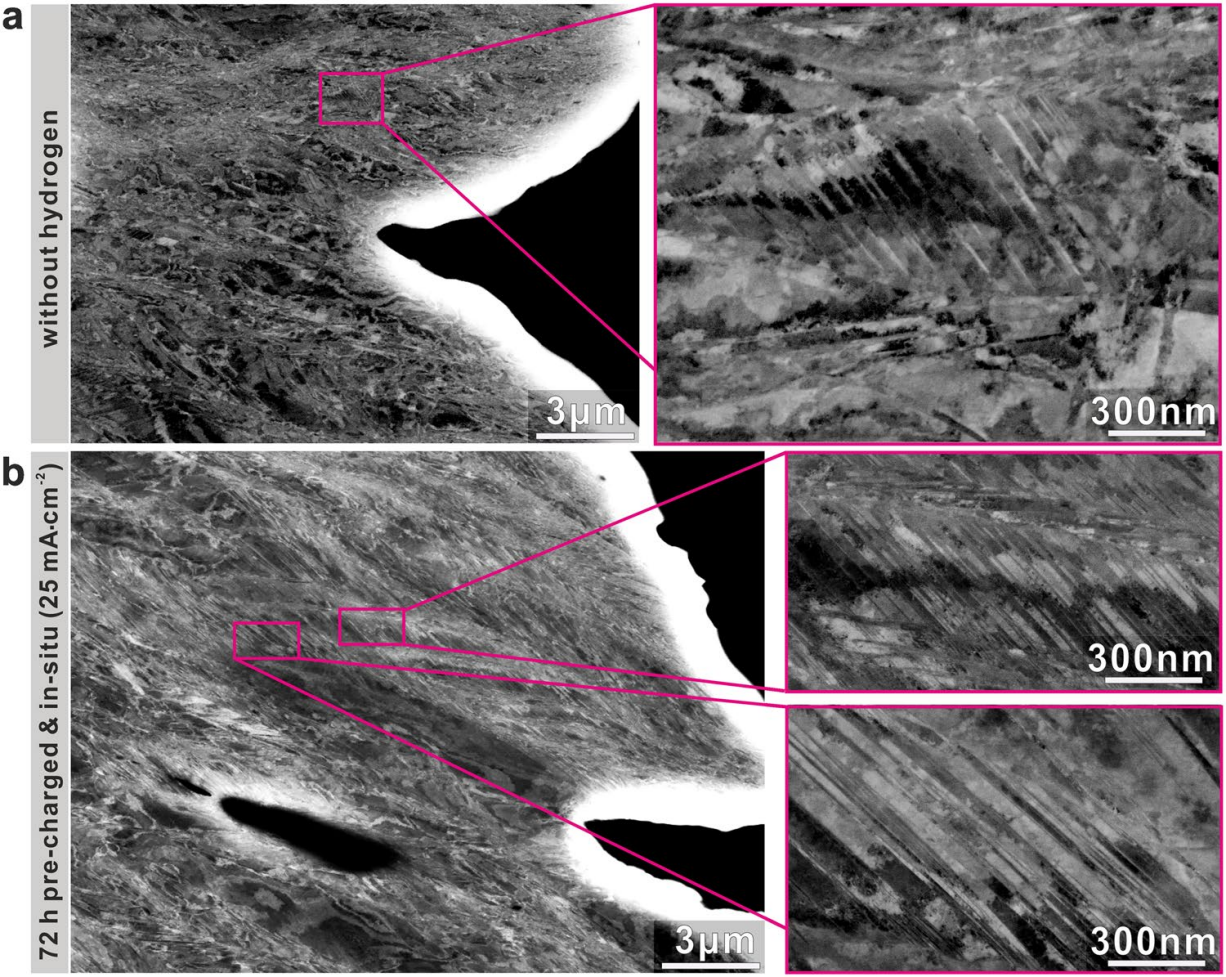
ECC images of deformation microstructures near the fracture surfaces of CoCrFeMnNi HEA samples without and with hydrogen. (**a**) A few deformation induced nanotwins formed in the fractured sample without hydrogen. (**b**) High density of nanotwins in the fractured sample pre-charged for 72 h at 25 mA·cm^−2^. ECC: Electron Channeling Contrast.

To reveal the micro-mechanisms behind the hydrogen induced joint increase in both, strength and ductility, we probed the deformation microstructures in the cross-sections of the CoCrFeMnNi HEA samples tensile tested under various hydrogen charging conditions. Figure 4 shows the typical ECC images of the microstructure near the fracture surfaces of the HEAs samples both with and without hydrogen charging. A few deformation nanotwins in the CoCrFeMnNi HEA sample without hydrogen were observed (Fig. 4a), which is consistent with a previous study which showed that deformation twining can occur in this HEA at relatively high strain levels at room temperature^36^. Interestingly, we observe that the density of nanotwins in the hydrogen alloyed HEA sample significantly increased compared to the hydrogen free alloy (Fig. 4 and Supplementary Fig. 5). For instance, as shown in Fig. 4b, the sample with a diffusible hydrogen concentration of 15.22 wt.ppm (72 h pre-charged at a current density of 25 mA·cm^−2^) shows a very high density of nanotwins compared to the HEA without hydrogen. We reveal the nanotwinning here by ECCI analysis^37^ using bulk samples to avoid any thin slice size and related effects which alter the stress and microstructure state of the material.

The equiatomic CoCrFeMnNi HEA has a relatively low intrinsic stacking fault energy (~19 mJ·m^2^)^38^, which explains the occurrence of mechanical twinning at room temperature without hydrogen charging. The strong increase in twinning density in the hydrogen alloyed HEA samples reveals that hydrogen reduces the stacking fault energy of the equiatomic CoCrFeMnNi HEA. This is consistent with the previous studies on the effect of hydrogen on the stacking fault energy in other f.c.c. structured alloys^39, 40^. Moreover, no other phase formed during the *in-situ* tensile testing with hydrogen charging according to the XRD results shown in Supplementary Fig. 6. These results also show that the increase in the density of nanotwins that we observe in the CoCrFeMnNi HEA leads to an associated increase in work hardening (Supplementary Fig. 3), which postpones the formation of geometric instabilities to higher strain values^41^, thereby increasing strength and ductility simultaneously.

The beneficial influence of nanotwins on strain hardening is commonly attributed to the “dynamic Hall-Petch” effect^29^, i.e. the formation of nanotwins leads to continuous grain fragmentation by the introduction of new interfaces which effectively reduces the dislocation mean free path, hence, causing further strengthening^42, 43^. Consistent with this, the strength and ductility of the CoCrFeMnNi HEA increased with the increase in the twinning density caused by the enhanced hydrogen concentration. However, during *in-situ* tensile testing, unfavorable mechanism caused by hydrogen may also become more significant with higher hydrogen concentration. For instance, hydrogen enhanced localized plasticity^16^ can occur in materials that are prone to planar slip, such as the here studied CoCrFeMnNi HEA^29^. This unfavorable mechanism was also observed in a gaseous hydrogen charged CoCrFeMnNi HEA^35^. Thus, a competition may exist between the beneficial nanotwining on the one hand and the unfavorable hydrogen embrittlement mechanisms on the other hand during deformation of the hydrogen charged HEA samples. Furthermore, considering the gradient in hydrogen concentration through the sample thickness, the very high concentration of hydrogen at the sample surface may result in initial surface cracks, thus reducing the overall strength and ductility of the bulk sample. The above mechanisms might be responsible for the fact that only small increments in ultimate strength and uniform elongation were observed in the HEA sample when apoproaching the highest diffusible hydrogen concentration (Fig. 2c).

## Conclusions

Our findings demonstrate that hydrogen can be utilized for the case of an equiatomic CoCrFeMnNi HEA to tune beneficial strengthening and toughening mechanisms rather than undergoing catastrophic failure due to hydrogen embrittlement. We show that hydrogen alloying with a proper concentration actually jointly increases both strength and ductility. By reducing the stacking fault energy and hence the phase stability of the CoCrFeMnNi HEA, hydrogen can cause a significant increase in the nano-twin density, thereby increasing the alloy’s work-hardening capability, and thus both strength and ductility. This breaks the previous preconception on the deleterious hydrogen effects which lasted for more than a century and provides new strategies for the future design of hydrogen tolerant materials with superior mechanical properties.

## Methods

### Materials preparation

The equiatomic CoCrFeMnNi HEA ingot with dimensions of 25 × 60 × 65 mm^3^ was cast in a vacuum induction furnace using pure metals with predetermined compositions (Co_20_Cr_20_Fe_20_Mn_20_Ni_20_, at.%). All the pure metals were cleaned properly and the carbon content was then controlled to be as low as possible to avoid the influence of interstitial carbon on the mechanical properties^44, 45^. Samples with dimensions of 10 × 25 × 60 mm^3^ machined from the original cast were subsequently hot-rolled at 900 °C to a thickness reduction of 50% (thickness changed from 10 to 5 mm). Homogenization was conducted at 1200 °C for 2 h in Ar atmosphere followed by water-quenching. To refine the grain size, samples were further cold-rolled to a thickness reduction of 60%, and subsequently annealed at a furnace temperature of 900 °C for 3 min in Ar atmosphere followed by water-quenching. Note that the true temperature that the samples actually reached during annealing might be lower than the furnace temperature (900 °C) due to the short annealing time.

### Microstructural and elemental characterization

The microstructure of the recrystallized alloy (grain-refined) was analyzed using various methods. X-ray diffraction (XRD) measurements were performed using an X-Ray equipment ISO-DEBYEFLEX 3003 equipped with Co Kα (λ = 1.788965 Å) radiation operated at 40 kV and 30 mA. Electron backscatter diffraction (EBSD) measurements were carried out by a Zeiss-Crossbeam XB 1540 FIB scanning electron microscope (SEM) with a Hikari camera and the TSL OIM data collection software. Electron channeling contrast imaging (ECCI) analyses of the deformation microstructures were performed on a Zeiss-Merlin instrument. The elemental distributions in the recrystallized alloy were investigated using energy-dispersive X-ray spectroscopy (EDS). The fracture morphology was observed by a Zeiss-Merlin instrument.

### Hydrogen charging and mechanical characterization

Hydrogen was introduced into the specimens by electrochemical charging with various current densities at ambient temperature (25 °C) in 0.1 M NaOH solution plus 0.05 g·L^−1^ CH_4_N_2_S at pH = 13. A platinum wire was used as the counter electrode. The samples were pre-charged for 12 h at 15 mA·cm^−2^, 72 h at 25 mA·cm^−2^, and 240 h at 100 mA·cm^−2^, respectively. Then the samples were continuously *in-situ* charged during the entire tensile testing to avoid the release of hydrogen. The tensile tests were conducted in an Instron tensile machine at the tensile rate of 1 × 10^−4^ S^−1^. Uniaxial tensile tests were conducted using specimens with thickness of 1.5 mm and gauge length of 10 mm. Three samples for each condition were tested to confirm reproducibility. The hydrogen desorption rates were measured by using a custom-designed UHV-based Thermal Desorption Analysis instrument in conjunction with a Mass Spectrometer detector set up (TDA-MS) from 25 °C to 800 °C, and the corresponding heating rate was 26 °C min^−1^. The diffusible hydrogen concentration was determined by measuring cumulative desorbed hydrogen from 25 °C to 600 °C.

## Electronic supplementary material

Supplementary information
