# Supplementary material for: Hydrogen enhances strength and ductility of an equiatomic high-entropy alloy
Source: Sci Rep. 2017 Aug 29;7:9892. doi: 10.1038/s41598-017-10774-4 (PMC5575320; doi:10.1038/s41598-017-10774-4)
Supplement: Supplementary file 1 — Supplementary information [file 41598_2017_10774_MOESM1_ESM.doc]

Supplementary information for

**Hydrogen enhances strength and ductility of an equiatomic high-entropy alloy**

Hong Luo, Zhiming Li, Dierk Raabe

Max-Planck-Institut für Eisenforschung, Max-Planck-Straße 1, 40237 Düsseldorf, Germany

Correspondence to: d.raabe@mpie.de (D. Raabe); zhiming.li@mpie.de (Z. Li)

**This part includes:**

Supplementary Table 1 and Figures 1 to 6

**Supplementary Table 1 | Average values of ultimate tensile strength and total elongation for the CoCrFeMnNi HEA under various in-situ hydrogen charging conditions at room temperature and at a strain rate of 1×10^-4^ s^−1^.**

|  | Ultimate tensile strength, (MPa) | Total elongation,  (%) |
| --- | --- | --- |
| #1: without hydrogen | 545 ± 4.1 | 68.4 ± 0.53 |
| #2: 12 h pre-charged & in-situ (15 mA·cm^-2^) | 571 ± 4.0 | 71.3 ± 0.61 |
| #3: 72 h pre-charged & in-situ (25 mA·cm^-2^) | 588 ± 3.1 | 71.5 ± 0.62 |
| #4: 240 h pre-charged & in-situ (100 mA·cm^-2^) | 576 ± 6.8 | 69.6 ± 0.66 |


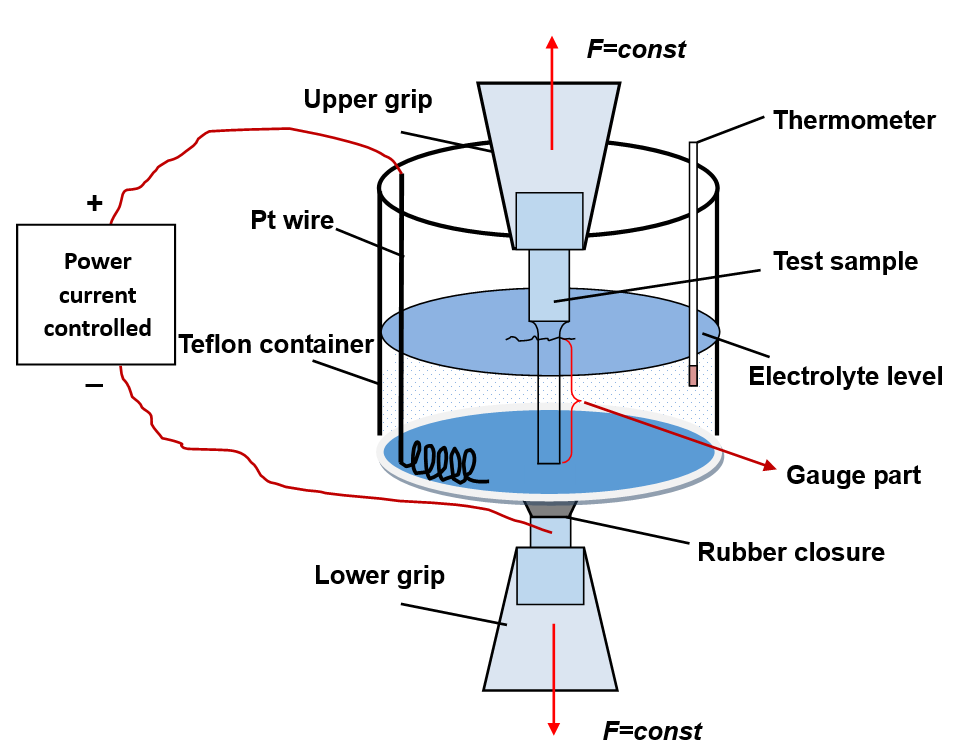


**Supplementary Figure 1 |** [**Schematic**](javascript:void(0);) [**diagram**](javascript:void(0);) **showing the in-situ hydrogen charging cell during tensile testing.**

**Supplementary Figure 2 | Hydrogen desorption rate curves of the samples with various hydrogen charging conditions.** The diffusible hydrogen concentration was determined by measuring cumulative desorbed hydrogen from 25 ^o^C to 600 ^o^C. The diffusible hydrogen concentrations corresponding to the four curves are: 0 wt.ppm (curve #1), 8.01 wt.ppm (curve #2), 15.22 wt.ppm (curve #3), and 33.25 wt.ppm (curve #4), respectively.

**Supplementary Figure 3 | Strain-hardening behavior corresponding to the tensile curves (Fig. 2c) of CoCrFeMnNi HEA under various in-situ hydrogen charging conditions.**

**
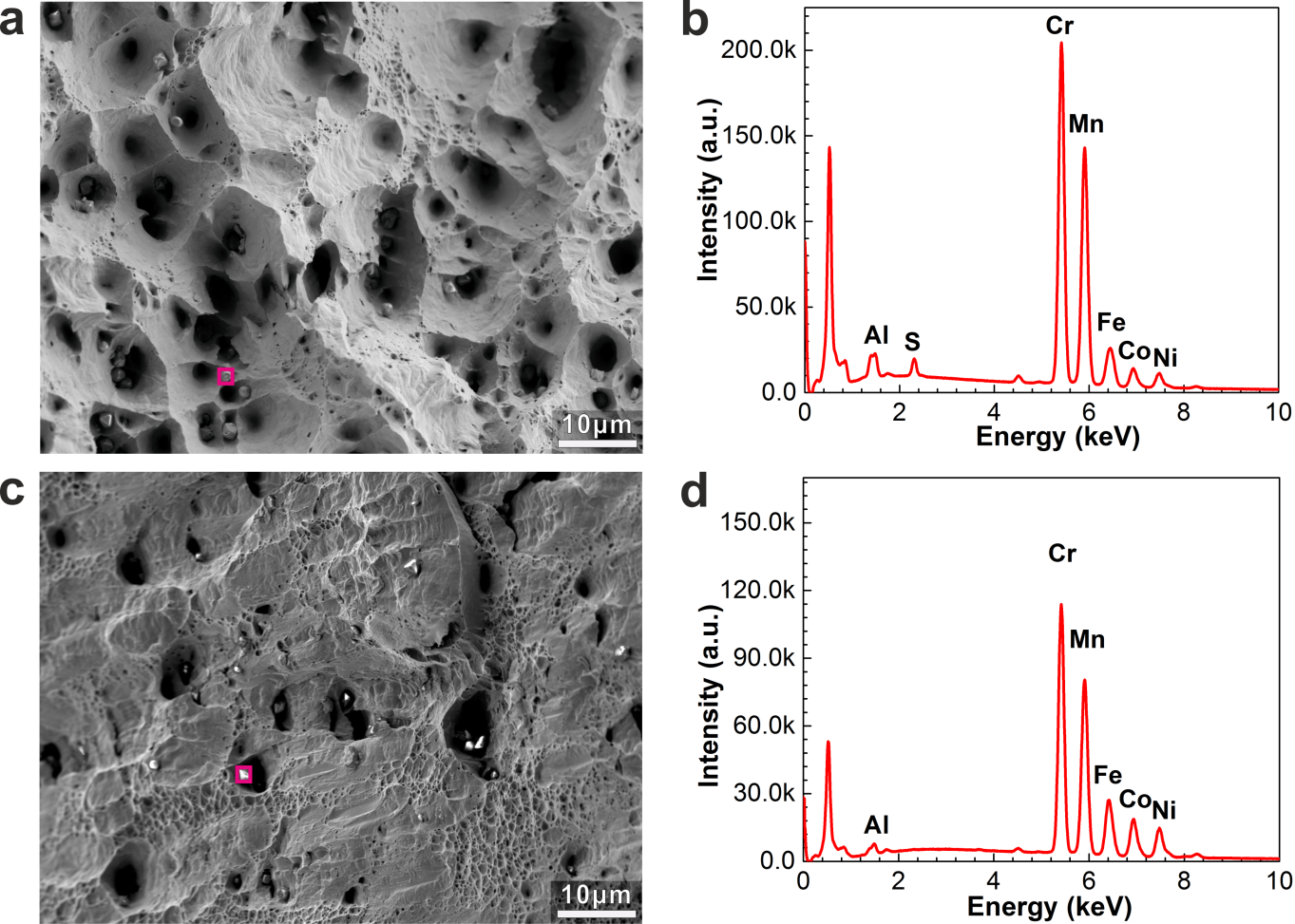
**

**Supplementary Figure 4 | EDS analysis of the particles inside the microvoids of the fracture surface. a**, **b**, Sample without hydrogen. **c**, **d**, Sample pre-charged for 72 h at 25 mA·cm^-2^. The results for both conditions show that the particles are enriched with Cr, Mn, S and Al.

**
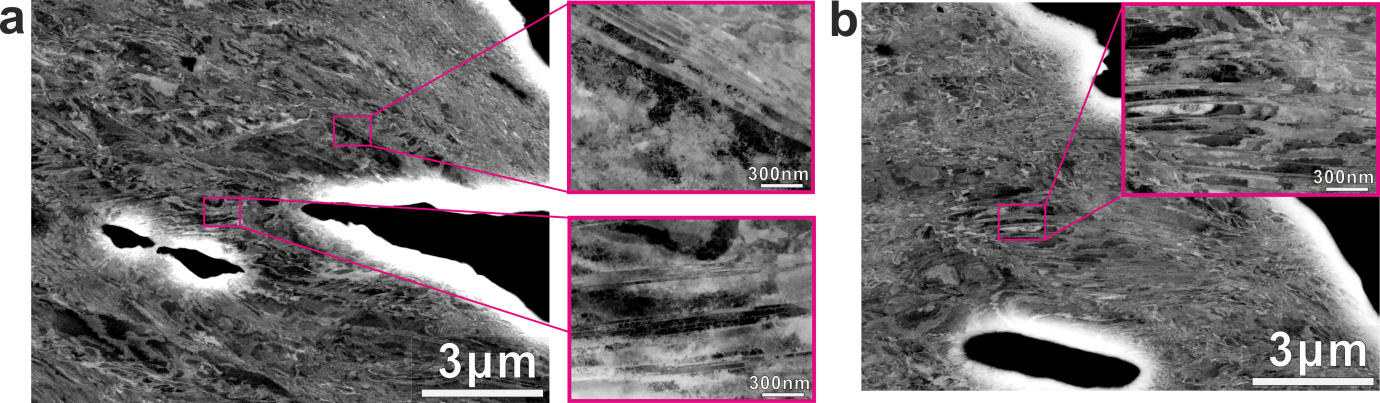
**

**Supplementary Figure 5 | ECC images of deformation microstructures near the fracture surfaces of CoCrFeMnNi HEA samples at ambient temperature. a**, Sample pre-charged for 12 h and in-situ tensile tested at 15 mA·cm^-2^. **b**, Sample pre-charged for 240 h and in-situ tensile tested at 100 mA·cm^-2^.

**
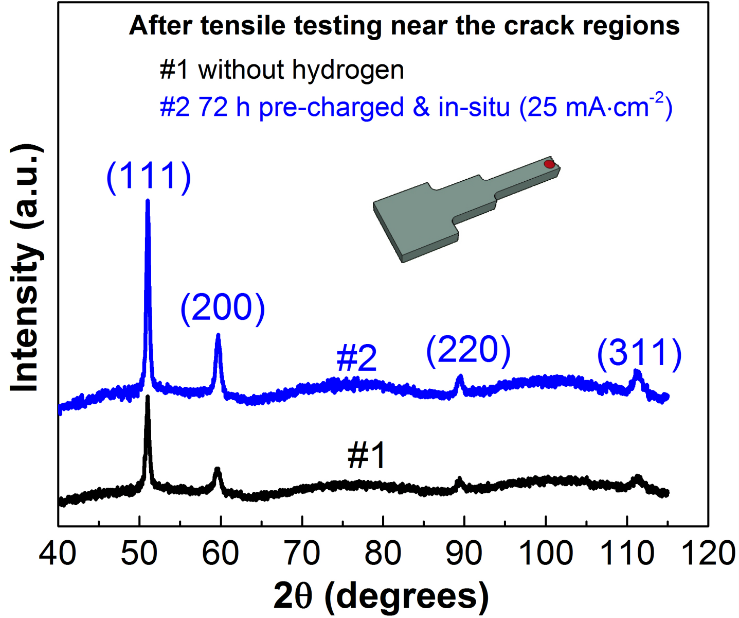
**

**Supplementary Figure 6 | XRD analysis of samples after tensile testing (near the fracture surface) without and with hydrogen.**
